# Australia: regulating genomic data sharing to promote public trust

**DOI:** 10.1007/s00439-018-1914-z

**Published:** 2018-08-16

**Authors:** Lisa Eckstein, Donald Chalmers, Christine Critchley, Ruthie Jeanneret, Rebekah McWhirter, Jane Nielsen, Margaret Otlowski, Dianne Nicol

**Affiliations:** 1 Centre for Law and Genetics, University of Tasmania Faculty of Law, Private Bag 89, Hobart, TAS 7001 Australia; 2 Faculty of Health, Arts and Design, Swinburne University, Melbourne, VIC 3122 Australia

## Abstract

The regulation of genomic data sharing in Australia is a confusing mix of common law, legislation, ethical guidelines, and codes of practice. Beyond privacy laws, which only apply to genomic data that meets the definition of personal information, the key regulatory lever is the National Health and Medical Research Council (NHMRC) *National Statement for Ethical Conduct in Human Research* (“National Statement”) (2007). Compliance with the *National Statement* is a requirement for institutions to apply to the NHMRC for funding, and includes—among other things—requirements for review of most genomic research by Human Research Ethics Committees. The sections of the *National Statement* specifying requirements for research with human genomic data are currently under review, including proposed new requirements addressing the return of genetic research findings and oversight of transfer agreements. Ensuring the willingness of Australians to donate their genomic information and participate in medical research will require clarification and harmonisation of the applicable regulatory framework, along with reforms to ensure that these regulations reflect the conditions necessary to promote ongoing public trust in researchers and institutions.

## Introduction

The regulatory environment for genomic data sharing in Australia is a confusing mix of common law, legislation, ethical guidelines, and codes of practice. Some understanding of the Australian legal system is required to better understand the various ways in which this regulatory environment operates in the context of genomic data sharing. Australia (or, more properly, the Commonwealth of Australia) has a complex legal system, befitting its status as a federation comprising six states and two territories. Section 51 of the Australian Constitution specifies that the federal legislature has the power to make laws with respect to specified issues. Powers that may be of particular relevance to this topic include external affairs, corporations, and interstate trade and commerce. The states and territories have the power to make laws with respect to all other issues; however, where there is overlap between a federal law and a state or territory law, the federal law will take priority. The Constitution is silent on matters such as privacy, healthcare, and medical research, resulting in a confusing array of federal alongside state and territory laws for many issues relevant to genomic data sharing.

State and territory courts have jurisdiction to hear the first-instance decisions relating to common law actions (like breach of contract, negligence, and trespass), as well as their own legislative instruments, although the High Court of Australia is the final court of appeal. Decisions of the High Court on common law actions are binding on all of the states and territories. However, decisions of state and territory courts that do not reach the High Court may cause differences in the interpretation of the common law between the states and territories. One example is the lack of uniformity across Australia about the status of a common law right to privacy (Australian Law Reform Commission 2014). Legislative differences among states and territories in a number of areas of law relevant to genomic data sharing, including privacy and human tissue legislation, also can create challenges for researchers.

The patchwork of Australia’s regulatory environment generates implications for public trust. Regulatory gaps create uncertain expectations as to how Australian institutions will protect the interests of research participants, especially those from vulnerable groups. Establishing such expectations is a key precursor to trust. In Australia, the absence of clear regulation and cohesive normative expectations arguably shifts the responsibility for protecting public trust to individual Human Research Ethics Committee (“HREC”) members, researchers, and clinicians. Fortunately, trust in these groups is relatively high (Bruce and Critchley 2015), but as the aftermath of biomedical scandals suggest, can easily be eroded. The potential implications go beyond those directly involved in the transgression (Dixon-Woods et al. 2011), and most importantly, can result in restrictive regulatory environments that could impede scientific discovery (Gillott 2014).

## Consent

Consent is an ethical and legal requirement for the collection, use, and sharing of much, but not all, genomic information and samples. For genomic information that meets the definition of “personal information” under privacy laws (discussed further below), consent is usually required.^1^ The sharing of human genomic information for research is also governed by ethical codes, principally the National Health and Medical Research Council (“NHMRC”), and *National Statement for Ethical Conduct in Human Research* (2007) (“*National Statement*”) (Australian Government National Health and Medical Research Council 2007).^2^

Unlike, for example, the US Common Rule, compliance with the *National Statement* is not a legal requirement. Rather, the key regulatory lever is institutional funding. Institutions such as public hospitals and universities can only apply to the NHMRC for funding if they declare that all research which they conduct complies with the *National Statement*. In their 2003 report on the protection of human genetic information (“*Essentially Yours*”), the Australian Law Reform Commission (“ALRC”) and the Australian Health Ethics Committee (“AHEC”)—one of the Principal Committees of the NHMRC—noted the extensive reach of the *National Statement* in governing Australian health and medical research, from direct provision of peer-reviewed health research funds and less linear connections. For example, research funded through the private sector but conducted at publicly funded institutions would have to be compliant (Australian Law Reform Commission 2003).^3^ Non-compliance with provisions of the *National Statement* by individual researchers, together with potential breaches of the Australian Code for the Responsible Conduct of Research (“*Australian Research Code*”) (Australian Government National Health and Medical Research Council et al. 2018), could form the basis of employment proceedings, a professional misconduct claim or an action in negligence for failure to meet the required standard of care for research (for example, *Medical Board of Australia v Melhuish* (Occupational Discipline) [2016] ACAT 29).

Consent requirements are captured in Chap. 2.2 of the *National Statement*, which specifies the “guiding principle” that “a person’s decision to participate in research is to be voluntary, and based on sufficient information and adequate understanding of both the proposed research and the implications of participation in it” (Australian Government National Health and Medical Research Council 2007). Furthermore, “[p]articipation that is voluntary and based on sufficient information requires an adequate understanding of the purpose, methods, demands, risks, and potential benefits of the research” (Australian Government National Health and Medical Research Council 2007). The *National Statement* does, however, build in provision for broad and blanket consent for the future use of data and tissue in research. In particular, it notes that consent may be:

- ‘specific’: limited to the specific project under consideration;
- ‘extended’: given for the use of data or tissue in future research projects that are:
  1. an extension of, or closely related to, the original project; or
  2. in the same general area of research (for example, genealogical, ethnographical, epidemiological, or chronic illness research);
- ‘unspecified’: given for the use of data or tissue in any future research (Australian Government National Health and Medical Research Council 2007, para 2.2.14).

Importantly, the *National Statement* clarifies that “The necessarily limited information and understanding about research for which extended or unspecified consent is given can still be sufficient and adequate for the purpose of consent”. When unspecified consent is sought, researchers should clearly explain to participants its terms and wide-ranging implications (Australian Government National Health and Medical Research Council 2007).

The *National Statement* also provides for waivers of consent in certain circumstances, including for some research involving human genomic information and samples. Before deciding whether to grant a waiver of consent, an HREC must be satisfied that it is impracticable to gain consent and that there is no known or likely reason that participants would not have consented if they had been asked (Australian Government National Health and Medical Research Council 2007). Chapter 3.5 of the *National Statement* details specific requirements for human genetic research, noting additional ethical issues based on the familial nature of the information and the potential for the information to provide predictive health information for participants and their family members. This chapter precludes researchers from sharing genetic material or related information in the absence of specific consent by participants or a judgment by an HREC that the provisions have been met for an extended or unspecified consent, or a waiver of consent (Australian Government National Health and Medical Research Council 2007).

Uncertainty exists, however, about the circumstances in which a waiver of consent should be granted (Otlowski and Nicol 2013). One common argument for waiver is the impracticability of seeking consent from participants who donated samples or information many years earlier. Yet, a 2002 study reported having recontacted 1409 out of 1494 persons who had donated blood for research purposes 11 years earlier. Of these, 1311 (93% of eligible participants) gave consent for future research using their blood samples. The authors concluded that it is feasible to obtain individual consent for genetic research many years after donation, and that people are highly willing to contribute to such research (Stegmayr and Asplund 2002). Other researchers, however, point to the time that it takes for researchers to contact participants, along with the potential distress that can be caused by inappropriate contact—for example, letters sent to participants who have since deceased (Furness and Nicholson 2004). In the absence of further clarification, it is likely that HREC positions on “impracticability” and other justifications for waivers of consent will vary.

The NHMRC has recently incorporated revisions to the *National Statement*, including an expansion of the ‘Human Genetics’ section (Chap. 3.5). The changes clarify circumstances in which specific consent is not required for human genomic research, being:

- if the data or information to be collected/generated/accessed/used was previously collected and either aggregated or rendered non-identifiable;
- if prior consent was provided under the scope of a research program that encompasses the proposed research project; or
- if prior consent was provided in the clinical context for research that encompasses the proposed research project (National Health and Medical Research Council 2016)

The changes also specify that researchers should not share genomic information unless such sharing is consistent with consent obtained either for the research project or for clinical purposes. Alternatively, an HREC may provide a waiver of consent where it considers that the requisite conditions have been met. Unlike present practices (Chalmers et al. 2014), an HREC also is required to approve any transfer agreement established for the sharing. These changes also include substantially more detailed and specific requirements for the return of genomic findings, including an obligation to have a process in place for the return of findings that are of proven validity and of health significance to the participant or relative, subject to participant consent (Australian Government National Health and Medical Research Council 2007).

Legal obligations to seek consent for the collection, use, and disclosure of genomic *samples*—as compared with genomic information—are set out in state and territory human tissue acts. Many of these are modelled on the seminal 1977 ALRC *Human Tissue Transplants* report, which set out draft legislation, including consent requirements for the removal of tissue for “therapeutic purposes or for medical or scientific purposes” [Australian Law Reform Commission 1977, Appendix 4 s 9(1)(e)]. Expressly excepted from consent requirements, however, was the removal or use of any tissue “from the body of a living person in the course of a procedure or operation carried out, in the interests of the health of the person, by a medical practitioner with the consent, express or implied, given by or on behalf of the person or in circumstances necessary for the preservation of the life of the person” [Australian Law Reform Commission 1977, Appendix 4 s 44(1)]. This exception has been implemented and retained in most state and territory human tissue acts, permitting the use of such samples in research. In New South Wales (NSW); however, amendments to the *Human Tissue Act 1983* (NSW) via the *Human Tissue and Anatomy Legislation Amendment Act 2003* (NSW) added a requirement for consent for the use of any tissue removed for clinical or other purposes for research.

Some research has been undertaken to investigate the wishes and expectations of Australians in relation to sharing genomic data for research, particularly in the context of biobanking. For instance, a deliberative democracy event in 2013 found participants to be broadly pragmatic, with a willingness to compromise on their concerns to facilitate better scientific outcomes. All but two supported a broad consent model for donation of human tissue and data (McWhirter et al. 2014). An earlier project involving semi-structured interviews reported a willingness among participants for donated specimens to be used “as the researchers saw fit”, and for waivers of consent for use of de-identified information. Reportedly, participants expressed concern that more rigid consent requirements could stifle valuable research (Lipworth et al. 2009). Recipients of clinical genetics cancer care also have expressed a willingness to waive active consent for storage of their de-identified information in a research database (Forrest et al. 2018). These studies are consistent with the international trends on participant willingness to share genomic information and samples for a broad spectrum of future research (Grady et al. 2015).

Researchers and clinicians involved in genomic data sharing must also be cognisant of the potential for group harm. In Australia, this is of particular concern for Aboriginal and Torres Strait Islander participants, for whom risks include genetic discrimination, racial stereotyping, cultural undermining, or harms associated with defining Aboriginality through genetics. In recognition of these concerns, HRECs may require community or group consent in addition to individual consents for genomic research involving Indigenous Australians. The NHMRC *Ethical Conduct in Research with Aboriginal and Torres Strait Islander Peoples and Communities: Guidelines for Researchers and Stakeholders* recognises that:

Decisions about participation in research may sometimes involve the whole community and not only individuals. Researchers may need to seek community consent as well as individual consent (Australian Government National Health and Medical Research Council 2018a).

Concerns about the potential harms specific to Indigenous participants have led to the exclusion of Indigenous Australians from much genomic research (McWhirter et al. 2015). This creates a problem, in that it limits the relevance of research findings to Aboriginal and Torres Strait Islanders and risks exacerbating existing health disparities.

In sum, the requirements to seek consent for participants in human genomic research in Australia are primarily derived from federal, state, and territory privacy laws, as well as the conditions for ethical approval under the National Statement. These frameworks allow researchers to obtain broad, as compared with specific, consent for the use of data or tissue in future research contingent on HREC approval and an explanation of the implications of such consent to potential participants. Waivers of consent are also permitted in situations in which an HREC deems that it is impracticable to gain consent and that there is no known or likely reason that participants would not have consented if they had been asked. The 2018 revisions to the National Statement will have the effect of better defining the circumstances in which specific consent is not required for human genomic research.

## Privacy

Australia has a complex patchwork of federal and state and territory privacy laws. This leads to areas of both over- and under-regulation, as well as some potential inconsistencies.

The federal public sector and private corporations are bound by the *Privacy Act 1988* (Cth) (“*Privacy Act*”). In addition, most Australian states and territories have enacted their own privacy statutes to govern information held by their public sectors and—in some cases such as Victoria and NSW—private health service providers in the state. In South Australia, the management of personal information is regulated through an administrative instruction (Government of South Australia 2016). In Western Australia, although some privacy principles are covered in the *Freedom of Information Act 1992* (WA), it applies solely to documents held by government agencies.

Under the federal *Privacy Act*, a regulated entity must not use or disclose personal information about an individual for a purpose other than the primary purpose of collection unless the individual has consented or another exception applies. Most relevant for the sharing of genomic information is an exception under s 16B(3) allowing the use or disclosure of health information if:

- the use or disclosure is necessary for research, or the compilation or analysis of statistics, relevant to public health or public safety;
- it is impracticable for the organisation to obtain the individual’s consent to the use or disclosure;
- the use or disclosure is conducted in accordance with the guidelines approved under section 95A for the purposes of this paragraph;
- in the case of disclosure—the organisation reasonably believes that the recipient of the information will not disclose the information, or personal information derived from that information.

Guidelines approved under s 95A of the *Privacy Act* provide a framework for HRECs, researchers, and others for the collection, use, or disclosure of personal health information without consent. These clarify that, for the use or disclosure to be considered “necessary” for research, an HREC must determine that “the outcome of the research activity would have an impact on or provide information about public health or public safety” and “the relevant purpose of the research activity cannot be achieved by the collection, use, or disclosure of de-identified data”. Similar exceptions are included in most state and territory privacy regimes, with a number adding the requirement that the disclosure will not be published in a form that identifies particular individuals. Notable exceptions are the South Australian administrative instruction and the Australian Capital Territory Act, which do not include a research exception from use and disclosure requirements.

The variation between federal and state and territory privacy laws creates challenges clarifying what law, or laws, apply to a given entity or activity. Most hospitals, research centres, and universities are established by state and territory legislation, making them subject to state and territory privacy regimes (Chalmers 2015). However, the practical realities of research funding and operations can complicate matters, particularly as the interface between research and clinical care is increasingly blurred in genomics (Berkman et al. 2014). For example, would a university researcher receiving private sector funding be governed by state or federal law? (Otlowski and Nicol 2013). Similarly, what regulations apply to research taking place in both public and private institutions?

In some states, private health sectors must comply with both state and federal laws, resulting in some potentially inconsistent obligations. An example is disclosure of genetic information to genetic relatives without consent. Under the *Privacy Act*, an organisation may disclose genetic information to a genetic relative without consent in circumstances when there is reasonable belief that disclosure is necessary to lessen or prevent a serious threat to the life, health, or safety of genetic relatives, and the use or disclosure is conducted in accordance with guidelines issued by the NHMRC (the s 95AA guidelines). In Victoria, private sector health service providers must also comply with the Health Privacy Principles set out in the *Health Records Act 2001* (Vic). This Act permits disclosure only in situations in which the sharing is necessary to lessen or prevent a serious *and imminent* threat to an individual’s life health and safety. Although an additional exception to the Victorian use and disclosure provisions where “the use or disclosure is required, authorised, or permitted… by or under law” appears to resolve this conflict, the inconsistency may still result in confusion for affected patients and researchers.

In the specific context of whole-genomic sequencing, there is uncertainty as to whether the information is “personal” and, therefore, subject to privacy requirements. This has not been directly tested in the courts. Under section 6 of the *Privacy Act*, personal information is defined as “information or an opinion about an identified individual, or an individual who is reasonably identifiable”. Clearly, human genome sequences directly linked to personal identifiers will fall within this definition. Much less certain is the extent to which whole-genome sequences satisfy the definition in the absence of specific identifiers. Researchers have shown the potential to deduce the individual identity of some persons based on their whole-genome sequences in combination with additional publicly available information (Gymrek et al. 2013). Although, at present, such approaches require considerable effort and ingenuity, along with the availability of certain genealogical information, their availability means that it is not fanciful for a court or a regulator to interpret a whole-genome sequence as being about “an individual who is reasonably identifiable”.

The 2018 revisions to the *National Statement* mentioned earlier impose some additional privacy requirements on researchers seeking to use or share genomic information, including an undertaking that they will not attempt to re-identify genomic material or information, nor permit such attempts by others. If researchers share genomic information for research, they also are required to make efforts to minimise the potential for re-identification (Australian Government National Health and Medical Research Council 2007). In addition, the proposed changes under the *Privacy Amendment (Re-identification Offence) Bill 2016* (Cth), designed to encourage the sharing of useful data, would make it an offence for any person to endeavour to re-identify personal information published on the basis of de-identification. At the time of writing, this Bill is before the Australian Senate (the upper house of federal parliament).

## Security measures

Security measures for storing and sharing human genome information must meet institutional requirements and conditions for approval by reviewing HRECs.

All human genomic research conducted at Australian universities and public research institutions must comply with the *Australian Research Code*. Section 1.6 sets out responsibilities on researchers to “follow proper practices for safety and security” and to maintain primary research materials and confidential research data in secure storage. Section 2.4 also obligates institutions to ensure the security and confidentiality of research data and primary research materials, including through developing and instituting policies for storage and access. Similar broad obligations are set out in the *National Statement*. Paragraph 5.5.5 requires that research is conducted in accordance with relevant security standards, and that institutions have responsibility for monitoring maintenance and security through regular reports provided by researchers.

In the case of personal information, additional security obligations arise under the *Privacy Act*. The most relevant of the Australian Privacy Principles (APPs) included in this Act is APP 11.1, which specifies that an entity that holds personal information “must take such steps as are reasonable in the circumstances to protect the information: from misuse, interference, and loss; and from unauthorised access, modification, or disclosure”. Notably, the principle may be breached even if no unauthorised access or misuse actually occurs. This raises two key questions in the context of human genomic information falling within the Act’s scope—when does an entity “hold” the information; and what steps are “reasonable” to protect against unauthorised access or misuse?

Section 6 of the *Privacy Act* clarifies that an entity will hold personal information if it has possession or control of a record that contains the information. The APP Guidelines issued by the Office of the Information Commissioner note that an entity might “hold” personal information in circumstances beyond physical possession, including where an entity has “the right or power to deal with the personal information, even if it does not physically possess or own the medium on which the personal information is stored” (Office of the Australian Information Commissioner 2015). This has particular pertinence for human genomic information stored on the cloud by the third parties (Office of the Australian Information Commissioner 2013).

In 2018, Australia implemented a Notifiable Data Breaches scheme through the *Privacy Amendment (Notifiable Data Breaches) Act 2017* (Cth). The scheme applies to all agencies and organisations with the existing security obligations under the *Privacy Act*. Notification obligations arise when there is unauthorised access to, disclosure of, or loss of, personal information and the access, disclosure, or loss is likely to result in serious harm to any individuals whose personal information is involved.

## Compatible processing/adequacy

The *Privacy Act* has extra-territorial application: through section 5B, it extends to any act done or practice engaged in outside Australia, by an agency, or by an organisation or operator with an Australian link, unless that act or practice is required by a foreign law. Section 16C deals with the cross-border transfer of personal information collected in Australia by an Australian entity. Where personal information is transferred by an Australian entity to an overseas recipient, and that recipient engages in conduct that would breach the APPs, the Australian entity will be taken to have engaged in the conduct and breached the APPs. APP 8.1 supplements s 16C of the Act, by providing that an Australian entity that discloses personal information about an individual to an overseas recipient, must take reasonable steps to ensure that the overseas recipient does not breach the APPs in relation to the information. The application of APP 8.1 is subject to a number of provisos. In particular, an Australian entity will not be required to take steps as required in APP 8.1 where it has a reasonable belief that a foreign law will provide an individual with protection should information about them be disclosed, or where the individual has consented to the disclosure after being made aware that APP 8.1 will not apply upon giving that consent.

The intention underlying s 16C and APP 8 was to introduce an accountability approach consistent with the Asia-Pacific Economic Cooperation (APEC) Privacy Framework (Asia-Pacific Economic Cooperation 2015). During 2017, the federal government undertook a public consultation to gauge support for Australian’s participation in the APEC Cross-Border Privacy Rules (CBPR) System (Australian Government Attorney-General’s Department 2017). The CBPR System “… requires participating businesses to develop and implement data privacy policies consistent with the APEC Privacy Framework” (Asia-Pacific Economic Cooperation). The consultation process resulted in a decision by the federal government to apply for Australia’s involvement in the system. Involvement will permit certification of businesses involved in cross-border data transfers, who are compliant with the requirements of the CBPR System.

What is not yet clear is whether Australia’s compliance with the CBPR system will provide adequacy under the European Union General Data Protection Regulation (“GDPR”). The *Privacy Act* shares many features with the GDPR in the context of protecting the private information of individuals (Office of the Australian Information Commissioner 2017). However, as a result of differences between the two regimes, Australia has yet to receive a ruling that its levels of data protection adequately conform with the GDPR, leaving the onus on Australian businesses operating in the EU to ensure individual compliance, including potentially by taking additional measures to meet GDPR requirements (de Sousa 2017).

## Oversight

In Australia, HRECs provide the principal form of oversight for genomic research. The *National Statement* requires HREC approval for all research that is more than low risk.^4^ This includes all human genetic research other than research on collections of non-identifiable data that carries only negligible risks to participants. The *Privacy Act* also requires HREC approval of research that may not comply with the standards imposed by the APPs (for example, an absence of participant consent to the sharing of personal information).

A prevailing question is whether most HRECs have adequate expertise to review the ethical aspects of human genomic research. Although HRECs are required to include members with the “current research experience relevant to the applications being reviewed”, there is no specific requirement for members with expertise in genomics. Without an effective mechanism for reviewing individual HREC membership and processes (Dodds 2002), it is impossible to ascertain whether they have sufficient scientific knowledge to review the vast array of genomic research projects coming before them. In 2003, *Essentially Yours* called on the NHMRC to develop and implement a quality improvement program for HRECs to promote consistent, efficient, and accountable review of human genomic research, including addressing the need for adequate HREC expertise (Australian Law Reform Commission 2003). This remains an ongoing concern.

A further challenge is the burden that the review process places on Australian researchers as against its concomitant benefits. Historically, HRECs were set up by individual research institutions, with approval required by each institution at which the proposed research was to be conducted. More recently, there have been moves towards shared reviews for multi-site research. New South Wales (NSW) was the first state to implement frameworks to support single ethical review of studies involving multiple public hospitals (Fraser et al. 2007). Under the NSW process, a single-certified HREC provides ethics approval for all designated sites. A separate governance review—incorporating, for example, consideration of the available budget and insurance and indemnity arrangements—is required to be conducted by each institution before a research project can commence at the site (Rush et al. 2017). In 2010, Queensland began to implement a process for single ethical review, with other states following in 2013 (Western Australia and South Australia) and 2015 (Victoria) (White et al. 2016).

These state-based schemes have been accompanied by efforts by the NHMRC to facilitate shared reviews at the national level. To this end, the National Approach to Single Ethical Review of Multi-Centre Research (formerly Harmonisation of Multi-Centre Ethical Review, HoMER) was implemented in 2007 (Johns et al. 2017). A central component of this scheme is national certification of participating institutions and their HRECs based on an independent assessment by the NHMRC (Australian Government National Health and Medical Research Council 2018b). Since November 2013, the Australian National Mutual Acceptance (NMA) Scheme has allowed certified HRECs to provide a single ethical review for multi-centre clinical trials conducted in public hospitals in certain states (initially Victoria, South Australia, Queensland and New South Wales, with Western Australia and the Australian Capital Territory joining more recently) (Australian Government National Health and Medical Research Council 2018c). In December 2015, the scope of the Scheme was expanded to include all multi-centre human research proposals.

These moves towards a single ethical review procedure promise a lessening of duplication and—through certification processes—greater quality assurance for ethical review of research seeking to share genomic information and samples. Nevertheless, gaps remain. Tasmania and the Northern Territory are not yet participants in the Australian NMA. Moreover, only full ethics applications are included in the NMA’s remit: the Scheme does not apply to low and negligible risk research, which may include some research using de-identified genomic information (Australian Government National Health and Medical Research Council 2018b). Importantly, in most states, private hospitals are not included in NMA single ethical review systems (White et al. 2016). Even when research projects are eligible for single ethical review, site-specific governance processes can impose considerable financial and time costs.

Challenges in implementation of streamlined ethics review in Australia are illustrated by the experiences of the Australian Pancreatic Cancer Genome Initiative (“APGI”), a research network aimed at cataloguing genomic abnormalities in pancreatic cancer patients through next-generation sequencing. The network recruited over 700 patients to its study between 2009 and 2013, requiring applications to 13 separate HRECs. Eight HRECs (61%) required a new project submission for full review, while five opted for expedited review based on prior approvals. Eight (61%) accepted the application using the NHMRC standard form, while the remainder required a customised form. The time taken to complete all submissions, including revisions and further communication, amounted to 74.5 working days, with an estimated overall cost of $60,471 (Johns et al. 2017). Other studies report similar experiences (De Smit et al. 2016; White et al. 2016; Rush et al. 2017).

## Future directions

Essentially Yours, completed in 2003, placed Australia as an early leader in the protection of human genetic information (Australian Law Reform Commission 2003). Fifteen years later, further work remains to ensure an effective and responsive framework for genomic information sharing. A promising development is the new revisions to the human genomics sections of the National Statement (Australian Government National Health and Medical Research Council 2007). The changes provide more specific and technologically aware advice to researchers seeking to share genomic information, including: obligations to minimise the potential for future re-identification; guidance on potential return of research findings; requirements for ethical review of transfer agreements for sharing genomic information. The next step will be ensuring the capacity of HRECs and others to implement the revisions, along with a streamlining of the oversight system more generally to ensure that single ethical review delivers net benefits.

The extensive and typically cross-border sharing arrangements that characterise modern genomic research also warrant an assessment of the interactions between multiple, often overlapping, legal regimes. An example here is the plethora of privacy laws which will govern some, but not all, sharing of genomic information depending on its degree of identifiability, whether the sharing involves public or private actors or some combination of the two, and the Australian states or territories in which the sharing is taking place. Questions also remain about the conditions under which HRECs should approve unspecified consent and waivers of consent for the sharing of genomic information, particularly as the amount of information available through, for example, whole-genome and whole-exome sequences allows for ever-greater inferences about personal, familial, and group characteristics.

Unanswered questions also remain in relation to public trust, especially for cross-border sharing and consent waivers. Public trust is a paramount consideration in maintaining Australians’ willingness to donate genomic information and participate in medical research (Critchley and Nicol 2017). Empirical research is only just beginning to shed light on preferences with regard to consent (Garrison et al. 2016), data storage (Sanderson et al. 2016), and sharing genetic information (Critchley et al. 2015). This work is, however, largely descriptive, unintegrated, and lacking examination of variation in views across jurisdictions. Unsurprisingly, findings are inconsistent: some indicate support for broad consent (Tomlinson et al. 2015) and data sharing (McGuire et al. 2011) to enable scientific discovery; others suggest the opposite, due to concerns about privacy breaches, for-profit exploitation and loss of control over how information may be used (Aitken et al. 2016).

There is a need to assess the impact of variation in contextual factors on public views of consent and cross-border data sharing (e.g., differences in privacy laws, ethical oversight, and the extent of public and private involvement). Recent work suggests the possibility that broad consent and waivers may be acceptable in jurisdictions with strong privacy protection and ethical oversight, in combination with strong trust in institutions and researchers (Critchley et al. 2017). Future research is needed to examine if, and under what conditions, Australians would accept consent waivers within the context of an understanding of the specific challenges facing genomic research and an awareness of Australian privacy and other protections.

## Footnotes

1 Under s 95A of the *Privacy Act 1988* (Cth), a waiver of consent may be approved by a Human Research Ethics Committee for the use of personal information in research relevant to public health or public safety where the public interest in the research is deemed to outweigh the public interest in the protection of privacy.

2 At the time of writing, parts of the National Statement are being revised including on human genetics and the use of human biospecimens.

3 Compliance with the National Statement also is a prerequisite for the use of unregistered therapeutic goods under the *Therapeutic Goods Regulations 1990* (Cth) and for certain uses of personal information under Commonwealth and state and territory privacy acts.

4 The *National Statement* defines “low risk” as “research in which the only foreseeable risk is one of discomforts.” In turn, discomforts are explained as including, for example, minor side-effects of medication, the discomforts relating to measuring blood pressure, and anxiety induced by an interview.
