# Correction to: Australia: regulating genomic data sharing to promote public trust

**DOI:** 10.1007/s00439-018-1930-z

**Published:** 2018-08-23

**Authors:** Lisa Eckstein, Donald Chalmers, Christine Critchley, Ruthie Jeanneret, Rebekah McWhirter, Jane Nielsen, Margaret Otlowski, Dianne Nicol

**Affiliations:** 1 Centre for Law and Genetics, University of Tasmania Faculty of Law, Private Bag 89, Hobart, TAS 7001 Australia; 2 Faculty of Health, Arts and Design, Swinburne University, Melbourne, VIC 3122 Australia

## Correction to: Human Genetics 10.1007/s00439-018-1914-z

This article was inadvertently published under a draft title.

The final title for this article is the following:

“Australia: Regulating Genomic Data Sharing to Promote Public Trust.”

The article Australia: regulating genomic data sharing to promote public trust, written by Lisa Eckstein, Donald Chalmers, Christine Critchley, Ruthie Jeanneret, Rebekah McWhirter, Jane Nielsen, Margaret Otlowski, Dianne Nicol, was originally published electronically on the publisher’s internet portal (currently SpringerLink) on 16 August 2018 without open access. With the author(s)’ decision to opt for Open Choice the copyright of the article changed on 5 September 2018 to © The Author(s) 2018 and the article is forthwith distributed under the terms of the Creative Commons Attribution 4.0 International License (http://creativecommons.org/licenses/by/4.0/), which permits use, duplication, adaptation, distribution and reproduction in any medium or format, as long as you give appropriate credit to the original author(s) and the source, provide a link to the Creative Commons license and indicate if changes were made.

The ​original ​article ​has ​been ​corrected.

